# MiR-539-3p inhibited chondrogenic differentiation in human adipose stem cells by targeting Sox9

**DOI:** 10.1186/s13018-022-03053-0

**Published:** 2022-03-18

**Authors:** Feng Qin, Fang Wang, Xiao-Ping Wang, Jie Chen, Feng-hua Zeng, Cui-Lan Sun, Jia-Cuo Peng Mao, Chun-Liang Li

**Affiliations:** 1 Department of Endocrinology, Qinghai University Affiliated Hospital, Xining, 810006 China; 2 Department of Gastroenterology, Qinghai University Affiliated Hospital, Xining, 810006 China; 3 Department of the Image Center, Qinghai University Affiliated Hospital, Xining, 810006 China; 4 Department of Anesthesia, Qinghai Provincial People’s Hospital, Xining, 810007 China; 5 Department of Clean Operating Room, Qinghai Provincial People’s Hospital, Xining, 810007 China; 6 Department of Orthopedic, Qinghai Provincial People’s Hospital, Chengdong District, No. 2 Gonghe Road, Xining, 810007 Qinghai China

**Keywords:** Adipose stem cells, Chondrogenic differentiation, miR-539-3p, Sox9

## Abstract

**Background:**

Mesenchymal stem cells (MSCs) have emerged as the attractive candidates for cell therapy for cartilage repair in clinical therapy of osteoarthritis (OA). MiR-539-3p was reported to differentially express during chondrogenic differentiation of adipose stem cells (ASCs) by miRNA microarrays. The aim of the study was to investigate the effects and underlying mechanisms of miR-539-3p on chondrogenic differentiation of ASCs.

**Methods:**

Human ASCs (hASCs) were obtained from liposuction and transfected with miR-539-3p mimic or inhibitor. Then, the cells were cultured in chondrogenic differentiation medium including transforming growth factor-β1 (TGF-β1).

**Results:**

Our results found that miR-539-3p was gradually down-regulated during chondrogenic differentiation of hASCs. MiR-539-3p overexpression inhibited TGF-β1-induced chondrogenic differentiation of hASCs, as supported by reducing the gene and protein expression of chondrogenic differentiation markers type II collagen alpha 1 (COL2A1), aggrecan (ACAN), and type II collagen. In contrast, miR-539-3p inhibitor significantly promoted the chondrogenic differentiation of hASCs. Dual luciferase reporter assay demonstrated that Sox9 was a direct target gene of miR-539-3p. The expression of SRY-box transcription factor 9 (Sox9) was up-regulated progressively over time during chondrogenic differentiation of hASCs. Additionally, Sox9 overexpression notably reversed chondrogenic differentiation of hASCs inhibited by miR-539-3p mimic, as demonstrated by the decreased expression of COL2A1, ACAN, and type II collagen.

**Conclusions:**

Altogether, miR-539-3p inhibited chondrogenic differentiation of hASCs by targeting Sox9. MiR-539-3p may have significant clinical applications for use as a targeted therapy of OA.

## Background

Osteoarthritis (OA) was the most common skeletal disorder in middle-aged and older people which resulted from articular cartilage degradation and imbalance of subchondral bone remodeling [1]. Repair of the cartilage defect and inhibition of cartilage degradation was most important for OA patients [2]. However, the majority of the drugs for the treatment of OA have been inadequate, as they only treated the symptoms of pain and inflammation [3]. Consequently more effective function-modifying therapeutic strategies would need to be introduced for the clinical treatment of OA.

In recent years, range of methods have been developed including osteochondral transplantation and autologous chondrocyte transplantation (ACT) [4]. However, ACT still faced challenges in treating cartilage defects, such as the paucity of the cell source and the damage to natural tissues caused by cell harvesting [5]. Recent researched on mesenchymal stem cells (MSCs) have provided a new and exciting opportunity for bone and cartilage tissue engineering [6, 7]. Thus far, MSCs have been isolated from bone marrow, periosteum, trabecular bone, adipose tissue, synovium, skeletal muscle, and deciduous teeth [8]. Among them, adipose stem cells (ASCs) have the advantage of being easy to obtain. ASCs possessed the capacity to differentiate into adipocytes, chondrocytes, osteoblasts, endothelial cells, cardiac myocyte and so on [8, 9]. New strategies have to center around promoting the chondrogenic potential of the chondrocytes using MSCs therapy.

MicroRNAs (miRNAs) were single-stranded RNAs of 19–23 nucleotides and were found in a wide variety of organisms [10, 11]. MiRNAs regulated gene expression in the post-transcriptional level through its complementary pair of target mRNAs, which leading to mRNA translation inhibition and degradation. MiRNAs were involved in the regulation of growth and development of various organisms, signal transduction, differentiation, and diseases [12]. Importantly, many miRNAs, such as miR-130a [13], miR-31a-5p [14], and miR-101-3p [15] were abnormally expressed in stem cells and played an important role in self-renewal and differentiation of stem cells by regulating the expression of certain key genes [16]. Previous miRNA microarrays analysis of hASCs during chondrogenic differentiation found that miR-539-3p was differentially expressed [17]. MiR-539-3p was recently reported to play a role in inhibiting proliferation and promoting apoptosis in a variety of cancers [18, 19], but there was no report on its role in regulating stem cell chondrogenic differentiation.

In this study, we explored the expression of miR-539-3p in the chondrogenic differentiation of ASCs. Furthermore, the study was performed to explore the biological functions and underlying mechanisms of miR-539-3p as a target gene expression inhibitor in the chondrogenic differentiation of ASCs.

## Materials and methods

### Isolation and culture of human ASCs (hASCs)

Human adipose was obtained from 5 healthy female donors, aged 33–42 years, during the abdominal liposuction procedure at the Qinghai Provincial People’s Hospital (Qinghai, China). Written informed consents were obtained from all donors. The research protocol was reviewed and approved by the Ethical Committee of Qinghai University Affiliated Hospital (SL-2021052). Adipose were digested with collagenase I to isolate ASCs, which were incubated in high-glucose DMEM with 1% fetal bovine serum (FBS) for primary culture. The third-passage ASCs were cultured in high-glucose DMEM supplemented with 1% FBS, 10 μg/L transforming growth factor-β1 (TGF-β1), 50 nmol/L ascorbate-2-phosphate, 6.25 mg/L insulin, and 1% penicillin. The appearance and generation of ASCs were observed under inverted microscope, and the differentiations to chondrocyte were verified by alcian blue staining and immunocytochemical staining.

### Cell transfection

NC mimic, miR-539-3p mimic, NC inhibitor, miR-539-3p inhibitor, pcDNA-NC, and pcDNA- SRY-box transcription factor 9 (Sox9) were designed and synthesized by RiboBio (Wuhan, China). Lipofectamine™ 3000 (L3000-008; Invitrogen, Grand Island, NY) transfection reagent was used for the transient transfection of according to the manufacturer's instructions.

### Cell proliferation assays

The 1–9 days growth curve of ASCs at the passage 3 was confirmed by cell counting kit-8 (CCK-8) assay. The cultures were supplemented with CCK-8 solution (Dojindo, Japan) and incubated for 1 h at 37 °C. Cell proliferation was measured at a wavelength of 450 nm by a microplate reader.

### Flow cytometry assays

Expression of ASC-related cell surface markers was evaluated by flow cytometry. Briefly, 5.0 × 10^5^ cells at the passage 3 were incubated with phycoerythrin (PE) conjugated monoclonal antibodies for human CD29, CD90, CD105, CD34, or CD45 (eBioscience, San Diego, CA, USA) for 1 h at 4 °C. The IgG isotype antibodies were used as negative controls for each experiment. After washing with PBS, cells were subjected to flow cytometric analysis using an FACS Canto II flow cytometer system (BD Biosciences).

### Alcian blue staining

Chondrogenic differentiation of ASCs was identified using alcian blue staining. ASCs were fixed using 4% paraformaldehyde for 10 min at room temperature. Then the cells were washed with PBS and performed using alcian blue solution (0.1% alcian blue, 0.1N HCl; Sigma-Aldrich, St. Louis, Missouri, USA) for 4 h at room temperature and images were captured.

### Immunofluorescence (IF) assays

ASCs (1.0 × 10^5^ cells) were fixed with 4% paraformaldehyde for immunofluorescence assay. For detection of collagen II expression, ASCs were blocked with 5% goat serum for 30 min and then incubated with primary anti- type II collagen (Abcam, USA, No. ab34712; 1/500) overnight at 4 °C. The slides were washed with PBS, secondary antibody was added and incubated for 1 h at room temperature before stained with 4',6-diamidino-2-phenylindole (DAPI; Invitrogen, Grand Island, NY; 1/1000). Finally, the stained samples were observed using a inverted microscope eclipse Ti (Nikon Instruments Inc., New York, USA).

### Quantitive Real-time reverse transcriptase-polymerase chain reaction (RT-qPCR)

RT-qPCR was performed to calculate the RNA expression of type II collagen alpha 1 (COL2A1), aggrecan (ACAN), Sox9, and miR-539-3p in ASCs (1.0 × 10^6^ cells) which were grown in 6-well cell culture plates. TRIzol protocol was used to extract total RNA from ASCs. 2000 ng RNA was then reverse transcribed into cDNA using GoScript™ reverse transcription system (Promega, USA). 1 µg cDNA was subjected to RT-qPCR using a SYBR Green Super mix (Bio-Rad, Hercules, CA, USA). The 2^‐ΔΔCT^ method was used to calculate the relative expression. β-actin was used as an internal control to normalize COL2A1, ACAN, and Sox9 expression. The levels of miR-539-3p expression were compared to that of the u6 control group. Primer sequences are shown in Table 1.

**Table 1:** Primers for quantitative RT-PCR

Gene	Forward (5′–3′)	Reverse (5′–3′)
COL2A1	CATTGCTGGTGCTCCCGGCT	ACCAGCATCACCAGGGCGAC
ACAN	GGAGGAGCAGGAGTTTGTCAA	TGTCCATCCGACCAGCGA AA
Sox9	AAGCTCTGGAGACTTCTGAACG	CGTTCTTCACCGACTTCCTCC
β-actin	CTCCATCGTCCACCGCAAATGCTTCT	GCTCCAACCGACTGCTGTCACCTTC
u6	CCAAGGTCATCCATGACAAC	TGTCATACCAGGAAATGAGC

### Dual-luciferase reporter assays

The wild type pSI-Check2-Sox9 (Sox9-3'UTR-Wt) and the mutant pSI-Check2-Sox9 (Sox9-3'UTR-Mut) recombinant dual luciferase reporter plasmid were designed and synthesized based on the binding region of miR-539-3p and Sox9 3'UTR sequence (Fubio Biological Technology Co., Shanghai, China). Sox9 Wt or Mut was co-transfected along with miR-539-3p mimic or NC into HEK293T cells by Lipofectamine™ 3000 (L3000-008; Invitrogen, Grand Island, NY). After 48 h, the luciferase activities were recorded with a plate style luminometer using Promega Dual-Luciferase system (Promega, Madison, Wisconsin, WI, USA).

### Western blot analysis

The total proteins of ASC lysates were collected RIPA buffer (Signaling Technology, Inc.). The protein concentration was determined using a BCA kit (Beyotime, Shanghai, China). Total protein (30 µg/sample) was separated via 10% SDS-PAGE and to nitrocellulose membranes. Use 5% skimmed milk powder to block the membrans. The corresponding protein antibodies were as follows: COL2A1 (Abclonal, China, No. A1560; 1/500), ACAN (Abcam, USA, No. ab3778; 1/1000), Sox9 (Abcam, USA, No. ab285966; 1/1000), and β-actin (Boster, Chian, No. BM0627; 1/1000). Then, the membrane washing was performed with Tris-buffered saline/0.1% Tween (TBST) and incubated for 1.5 h with a HRP Goat anti-Rabbit IgG (Abcam, No. ab6721; 1/1000). The band visualization was carried out using the ECL system (Thermo Scientific, Rockford, IL, USA). β-actin used as internal control.

### Statistical analysis

For isolation and identification of hASCs, the experiments were performed 5 times independently, using ASC from 5 different people. For additional cell study, each experiment was repeated at least 3 times independently. The data were represented as means ± standard deviation (SD). Statistical analysis was performed with the SPSS software (version 19.0, SPSS Inc., Chicago, IL, USA). One-way analysis of variance (ANOVA) followed by Tukey's post hoc test and Student's unpaired t test were used for comparison between groups. *P* value < 0.05 was considered statistically significant.

## Results

### Isolation and identification of ASCs

**Fig. 1:**
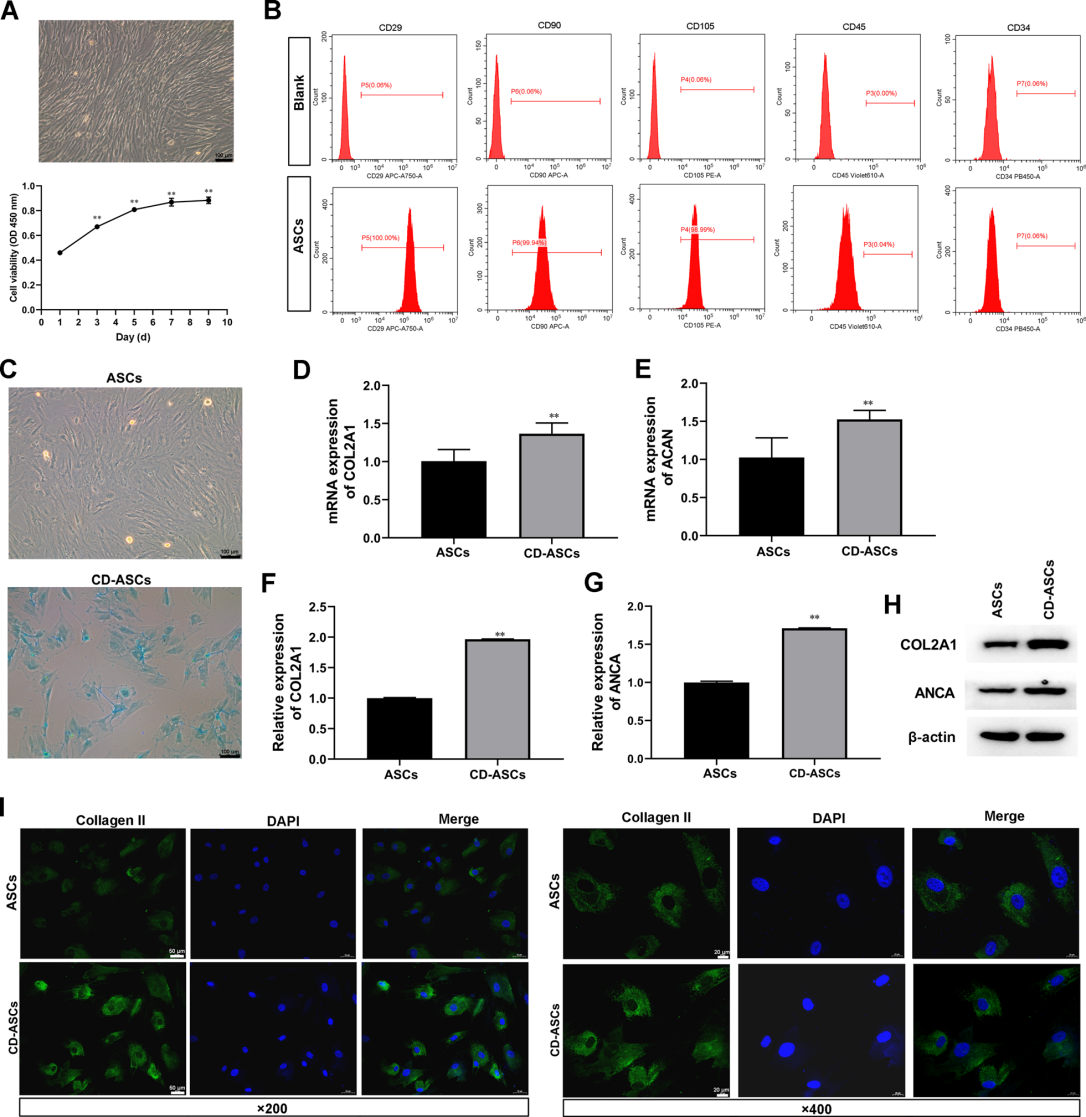
Classification of human adipose stem cells (hASCs). **A** hASCs exhibited a representative spindle-like morphology. Scale bar: 100 μm. The cell proliferation of hASCs was tested by CCK-8 assay. ^**^P < 0.01 (VS. 1 day). **B** Flow cytometric analysis of characteristic cell surface markers of hASCs (CD29, CD90, CD105, CD45, and CD34). **C** Alcian blue staining of hASCs. Scale bar: 100 nm. **D**–**E** The mRNA levels of COL2A1 and ACAN were quantified by RT-qPCR. **F**–**H** The protein levels of COL2A1 and ACAN were measured using western blot. β-actin expression levels were detected as an endogenous control. ^**^P < 0.01 (VS. ASCs). **I** Immunofluorescence assay of Type II collagen. Type II collagen fluorescence (green) and DAPI (blue). Scale bar: 50 μm and 20 μm. The experiment was repeated independently 5 times. The data are presented as mean ± standard deviation (SD)

ASCs were isolated from human abdominal adipose tissue as described in the Methods section. At passage 3 (P3), ASCs were identified and used in subsequent experiments. After reaching 80–90% confluence, cells adopted a spindle-like shape (Fig. 1A). And CCK-8 assay showed that the cell viability of ASCs was significantly increased during 10 days of continuous culture (Fig. 1A). Meanwhile, ASCs were characterized by flow cytometry after staining surface markers. These cells were found to be positive for CD29, CD90, and CD105, but did not express CD45 and CD34 (Fig. 1B). hASCs were cultured in chondrogenic differentiation medium that were evaluated by alcian blue staining (Fig. 1C). The resulted showed that alcian blue staining was positive (Fig. 1C). Moreover, the expression of chondrogenic differentiation markers, such as COL2A1 and ACAN were analyses to confirmed chondrogenesis. The mRNA and protein levels of COL2A1 and ACAN were increased in chondrocyte-ASCs (CD-ASCs, Fig. 1D–H, and Type II collagen in IF staining was appeared positive (Fig. 1I).

### MiR-539-3p regulated ASC chondrogenic differentiation

**Fig. 2:**
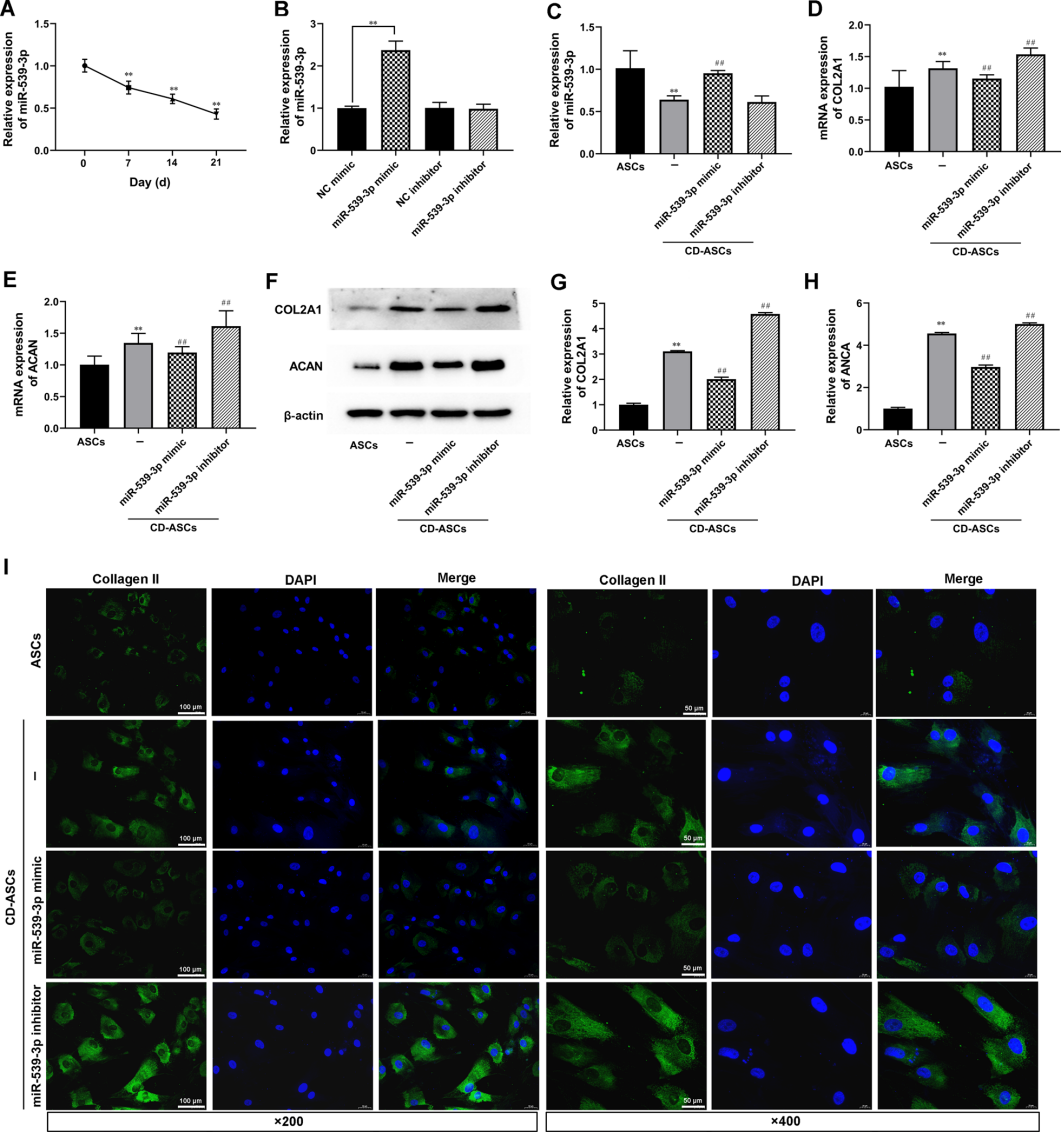
MiR-539-3p regulated ASC chondrogenic differentiation. hASCs were transfected with miR-539-3p mimic or miR-539-3p inhibitor, which were cultured in chondrogenic differentiation medium. hASCs cultured in high-glucose DMEM with 1% fetal bovine serum (FBS) were used as control. **A**–**C** The expression levels of miR-539-3p in hASCs or chondrogenic differentiation hASCs (CD-ASCs) were detected by RT-qPCR. Gene expression levels of COL2A1 and ACAN in hASCs or CD-ASCs were determined by RT-qPCR (**D** and **E**) and western blot (**F**–**H**). β-actin expression levels were detected as an endogenous control. ^**^P < 0.01 (VS. Day 0/NC mimic/ASCs), ^##^*P* < 0.01 (VS. CD-ASCs). (I) Immunofluorescence assay of Type II collagen. Type II collagen fluorescence (green) and DAPI (blue). Scale bar: 100 μm and 50 μm. The experiment was repeated at least 3 times independently. The data are presented as mean ± standard deviation (SD)

As shown in Fig. 2A, the expression of miR-539-3p gradually decreased at day 7, 14 and 21 of ASC chondrogenic differentiation, implying that miR-539-3p expression may relate with ASC chondrogenic differentiation. To confirm whether miR-539-3p modulates chondrogenic differentiation in ASCs, miR-539-3p mimic, miR-539-3p inhibitor, and their negative controls were designed and synthesized by RiboBio to stablely overexpress miR-539-3p and inhibit the function of miR-539-3p, respectively (Fig. 2B and C). We tested COL2A1, ACAN, and Type II collagen expression to explore the effect of miR-539-3p on hASC chondrogenic differentiation. As shown in Fig. 2D and E, the down-regulated COL2A1 and ACAN mRNA expression were observed in miR-539-3p mimic transfected CD-ASCs. And the treatment of miR-539-3p inhibitor showed the opposite results (Fig. 2D and H). Meanwhile, COL2A1 and ACAN were lower expressed in miR-539-3p mimic transfected CD-ASCs and higher expressed in miR-539-3p inhibitor treated CD-ASCs (Fig. 2F–H). IF assay showed that miR-539-3p overexpression decreased Type II collagen expression and miR-539-3p inhibitor increased Type II collagen expression in CD-ASCs. These results demonstrated that miR-539-3p diminished the chondrogenic differentiation of ASCs.

### Sox9 is a target gene of miR-539-3p

**Fig. 3:**
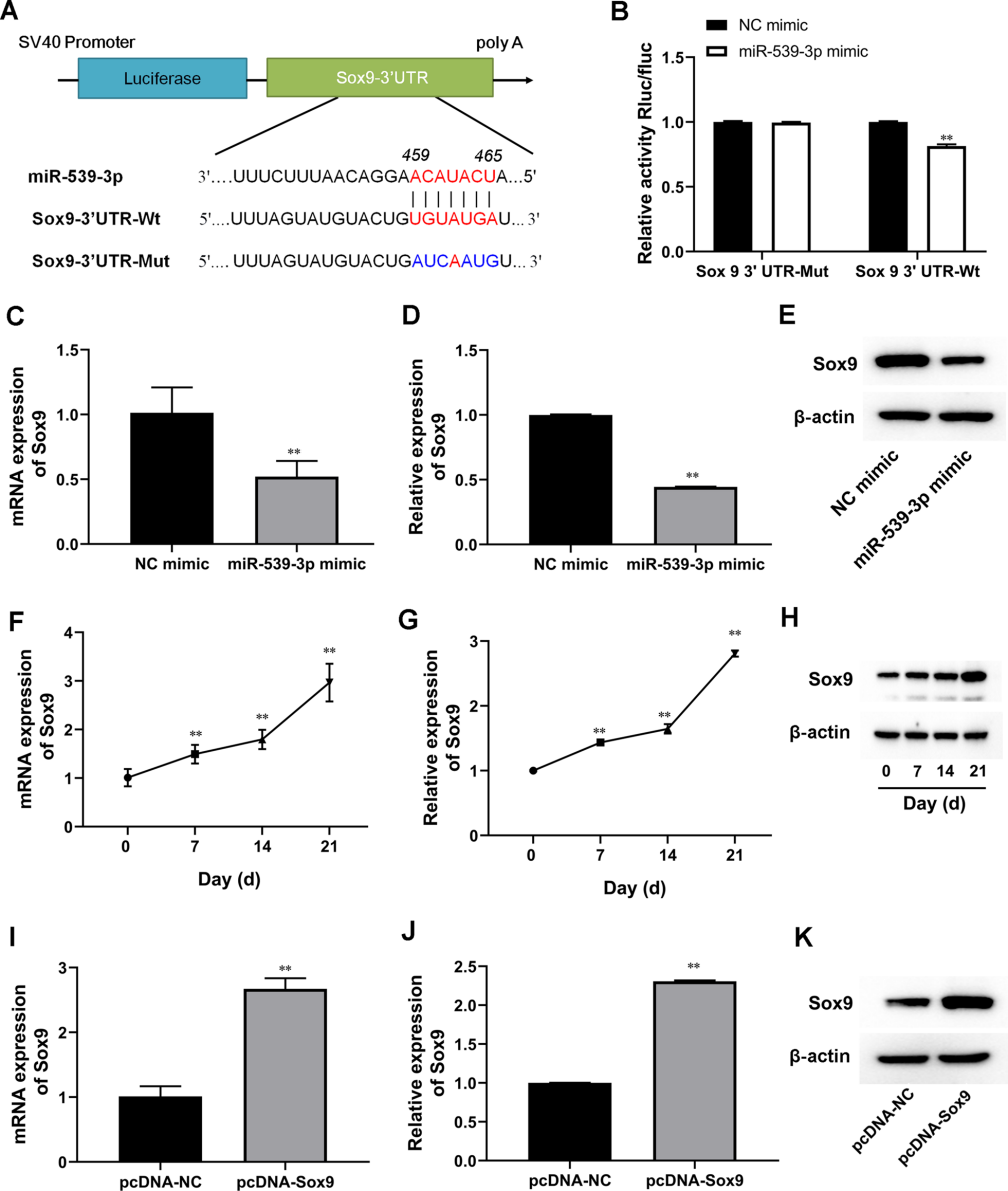
Sox9 is a target gene of miR-539-3p. **A** Alignment of miR-539-3p and the 3′-UTR of Sox9, a potential miR-539-3p target. **B** A luciferase reporter carrying the 3′-UTR of wild-type (Sox9 3′UTR-WT) or mutant (Sox9 3′UTR-MUT) Sox9 was introduced into HEK-293T cells along with NC mimic or miR-539-3p mimic. **C**, **F**, and **I** The expression level of Sox9 in hASCs was determined by RT-qPCR. **D**, **E**, **G**, **H**, **J**, and **K** The protein expression levelof Sox9 in hASCs was detected by western blot. β-actin expression levels were detected as an endogenous control. ^**^P < 0.01 (VS. 0 Day/NC mimic/pcDNA-NC). The experiment was repeated at least 3 times independently. The data are presented as mean ± standard deviation (SD)

Subsequently, the putative binding site of miR-539-3p to the 3′-UTR of Sox9 was predicted using the TargetScan and Starbase v2.0 (Fig. 3A). Luciferase assay demonstrated that the luciferase activity of wild type pSI-Check2-Sox9 was attenuated by miR-338-3p inHEK293T cells (Fig. 3B). Conversely, no significant differences were observed in the luciferase activity of the mutant pSI-Check2-Sox9 (Fig. 3B). Our results further showed that miR-539-3p mimic significantly inhibited Sox9 expression (Fig. 3C–E). Moreover, the mRNA and protein levels of Sox9 were increased with time following chondrogenic differentiation in ASCs (Fig. 3F–H). These findings indicated that miR-539-3p suppressed Sox9 expression by directly binding to the 3'-UTR of Sox9.

### Sox9 overexpression promoted ASC chondrogenic differentiation by blocking the effect of miR-539-3p mimic

**Fig. 4:**
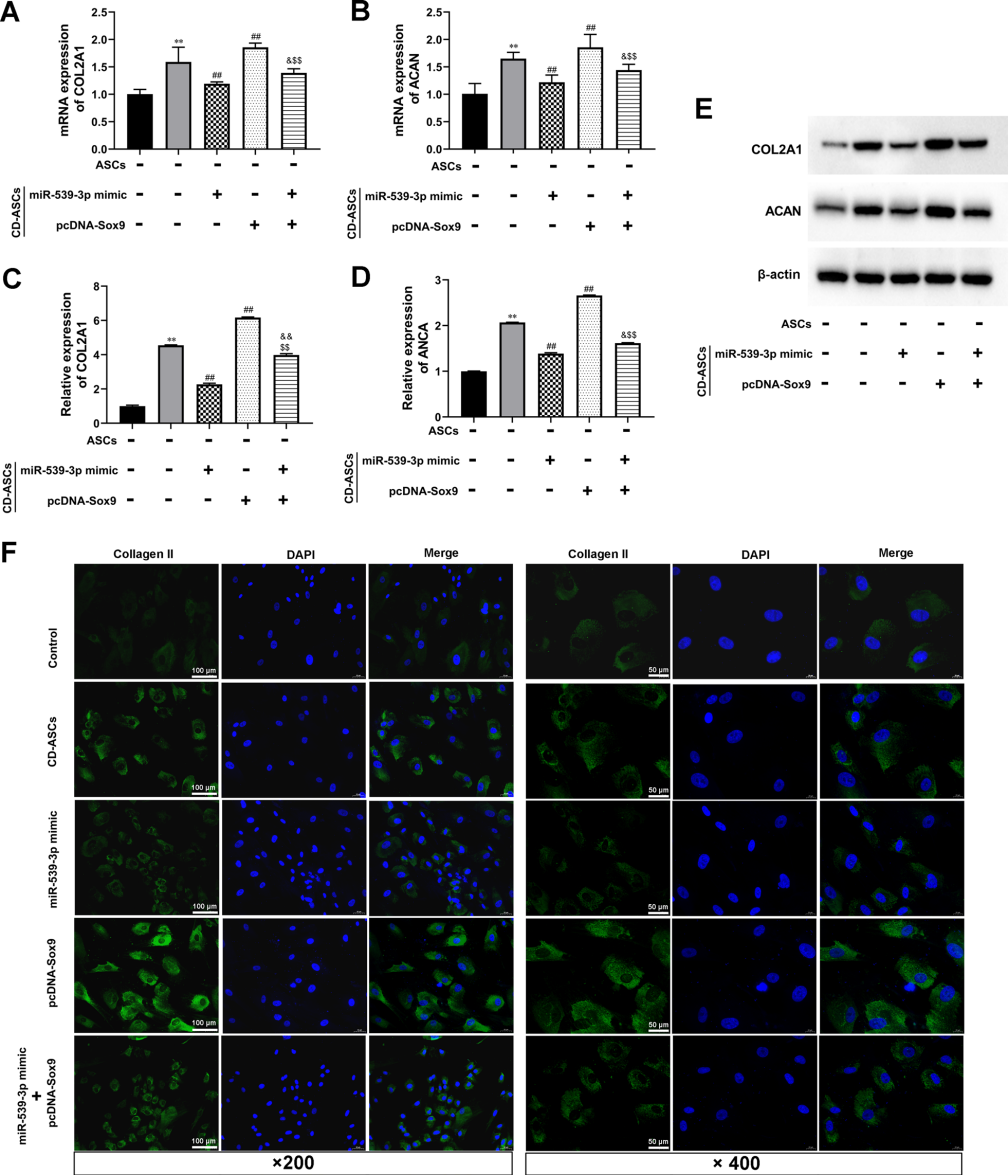
Sox9 overexpression promoted hASC chondrogenic differentiation by blocking the effect of miR-539-3p mimic. hASCs were transfected with miR-539-3p mimic or/and pcDNA-Sox9, which were cultured in chondrogenic differentiation medium. hASCs cultured in high-glucose DMEM with 1% fetal bovine serum (FBS) were used as control. Gene expression levels of COL2A1 and ACAN in hASCs or CD-ASCs were determined by RT-qPCR (**A** and **B**) and western blot (**C**–**E**). β-actin expression levels were detected as an endogenous control. ^**^P < 0.01 (VS. ASCs), ^##^*P* < 0.01 (VS. ASCs), ^&^*P* < 0.05 (VS. miR-539-3p mimic), ^&&^*P* < 0.05 (VS. miR-539-3p mimic), ^$$^*P* < 0.01 (VS.pcDNA-Sox9). (I) Immunofluorescence assay of Type II collagen. Type II collagen fluorescence (green) and DAPI (blue). Scale bar: 100 μm and 50 μm. The experiment was repeated at least 3 times independently. The data are presented as means ± standard deviation (SD)

To verify the regulatory function of Sox9 on miR-539-3p-mediated chondrogenic differentiation, Sox9 overexpression vector (pcDNA-Sox9) was synthesized and co-transfected with miR-539-3p mimic into CD-ASCs. As shown in Fig. 3I–K, the mRNA and protein levels of Sox9 were both enhanced by pcDNA-Sox9. After chondrogenic differentiation was induced in media for 21 days, Sox9 overexpression reversed miR-539-3p-reduced COL2A1 and ACAN expression compared with the alone transfection of miR-539-3p mimic group. IF detection also showed that the expression of Type II collagen was decreased by miR-539-3p mimic, which was significantly blocked by Sox9 overexpression. These data indicated that miR‑490‑5p inhibited the chondrogenic differentiation of ASCs by targeting Sox9 and decreasing Sox9 expression (Fig. 4).

## Discussion

In the present study, the down-regulated of miR-539-3p was observed on day 7, 14 and 21 during TGF-β1-induced chondrogenic differentiation of ASCs. Furthermore, bioinformatics prediction and dual-luciferase reporter assays verified Sox9 was a target gene of miR-539-3p. Overexpression of miR-539-3p inhibited the expression of chondrogenic differentiation markers, such as COL2A1 and ACAN, as well as Type II collagen expression. Sox9 overexpression significantly reversed the anti-chondrogenic effect of miR-539-3p in ASCs.

MiRNAs regulate gene expression at the posttranscriptional level in many developmental and metabolic processes. Emerging evidence demonstrates that miRNAs also play an essential role in stem cell self-renewal and differentiation [20, 21]. Some miRNAs are abnormally expressed in stem cells, control stem cell self-renewal, and differentiation through negatively regulating the expression of certain key genes in stem cells [22]. The expression profile of miRNAs during chondrogenic differentiation of hASCs showed that 8 miRNAs were down-regulated and 12 miRNAs were up-regulated [17]. Among them, miR-490-5p facilitated the chondrogenic differentiation of hASCs by targeting PITPNM1 3'UTR and regulating PI3K/AKT axis [23]. MiR-1307-3p overexpression suppressed the transition of hASCs from chondrogenesis to osteogenesis, and further mechanistic evaluation showed that it functioned by targeting BMPR2 and inhibiting Smad5/8 signaling pathway [24]. An additional study found that miR-194 was a novel modulator of hASCs-mediated chondrogenesis by targeting Sox5 [25]. MiRNA let-7i-3p inhibited the chondrogenic differentiation of hASCs, and inhibiting the function of let-7i-3p activated the Wnt/β-catenin pathway. Our RT-qPCR data indicated that miR-539-3p was obviously decreased during process of chondrogenic differentiation of hASCs. Furthermore, miR-539-3p mimic inhibited hASC chondrogenic differentiation by reduced the expression of chondrogenic markers Type II collagen, COL2A1, and ACAN. The inhibited of miR-539-3p function showed the opposite trends. These data demonstrated that miR-539-3p functions as a suppressor during chondrogenic differentiation of hASCs. Several previous reports of miR-539-3p were mostly focused on regulating cancer progression. MiR-539-3p was reported to promote the proliferation and invasion of epithelial ovarian cancer and inhibit the progression of gastric cancer and colon cancer [18, 19, 26]. The mechanism of miR-539-3p-mediated tumorigenesis was targeted SPARCL1 [18], RNF2 [27], CTBP1 [19], or CDK14 [26] and inhibited the expression of these proteins in tumor cells. In addition, another study revealed that in a model of ischemia–reperfusion (I/R) cardiac injury, miR-539-3p enhanced infarction area and stimulated oxidative stress, myocardial cell apoptosis, and reduced cardiac function recovery via targeting ErbB4 [28]. Accordingly, to the best of our knowledge, this study was the first to focus on miR-539-3p function in chondrogenic differentiation of hASCs. Further in vivo functional analyses will be needed to clarify the potential of miR-539-3p to treat OA with stem cell transplantation.

Sox9 was an important transcription factor for chondrocyte differentiation and played an important role in the transformation of stem cells into chondrocytes and in the process of chondrogenesis. Previous report showed that a denovirus-mediated Sox9 overexpression vector delivery induced chondrogenic differentiation and inhibited osteogenic and adipogenic differentiation of hMSCs [29]. The study by Magali Cucchiarini found that application of recombinant adeno-associated virus (rAAV) Sox9 vector significantly treated rabbit knee articular cartilage defects and delayed premature terminal differentiation in the newly formed cartilage [30]. More importantly, in a rabbit model with full-thickness cartilage defects, bone marrow mesenchymal stem cells (BMSCs) transfected with Sox9 gene adenovirus were loaded on polyglycolic acid (PGA) scaffolds to repair chondral defects. The results revealed that this Sox9-BMSCs-PGA scaffold composition promoted cartilage matrix formation and chimerism of new cartilage with surrounding tissue [31]. Further, it was known that a variety of miRNAs, such as miR-30b [32], miR-495 [33], and miR-574-3p [34] could negatively regulate Sox9 in chondrogenic differentiation of stem cells. In the present study, Sox9 was identified as a target gene of miR-539-3p by luciferase reporter assay, which plays a role in promoting the chondrogenic differentiation of hASCs. Previous studies also showed that Sox9 target genes included COL2Al, COL11Al, COL9A2, Aggrecan (Agc1), cartilagelink protein (CRTL1), and so on [35]. We found that Sox9 overexpression violently reversed the inhibitory effect of miR-539-3p mimic on hASCs executing the chondrogenic differentiation program, indicated by increasing expression of Type II collagen, COL2A1, and ACAN.

## Conclusion

Our in vitro study using hASCs is the first demonstration that miR-539-3p was down-regulated during chondrogenic differentiation. Our findings are also found miR-539-3p inhibited chondrogenic differentiation of hASCs by targeting Sox9. Our results may provide a potential reference target for the treatment of OA. Further experimentation will be needed to investigate whether miR-539-3p inhibitor‑modified ASCs could also promote chondrogenesis in vivo.

## Data Availability

The datasets used or analyzed during the current study are available from the corresponding author on reasonable request.

## Abbreviation

MSCs: Mesenchymal stem cells
OA: Osteoarthritis
ASCs: Adipose stem cells
TGF-β1: Transforming growth factor-β1
COL2A1: Type II collagen alpha 1
ACAN: Aggrecan
Sox9: SRY-box transcription factor 9
ACT: Autologous chondrocyte transplantation
miRNAs: MicroRNAs
IF: Immunofluorescence
RT-qPCR: Quantitive Real-time reverse transcriptase-polymerase chain reaction
Agc1: Aggrecan
CRTL1: Cartilagelink protein
